# collectNET: a web server for integrated inference of cell–cell communication network

**DOI:** 10.1093/database/baae098

**Published:** 2024-09-16

**Authors:** Yan Pan, Zijing Gao, Xuejian Cui, Zhen Li, Rui Jiang

**Affiliations:** Ministry of Education Key Laboratory of Bioinformatics, Bioinformatics Division at the Beijing National Research Center for Information Science and Technology, Center for Synthetic and Systems Biology, Department of Automation, Tsinghua University, FIT 1-107, Beijing 100084, China; Ministry of Education Key Laboratory of Bioinformatics, Bioinformatics Division at the Beijing National Research Center for Information Science and Technology, Center for Synthetic and Systems Biology, Department of Automation, Tsinghua University, FIT 1-107, Beijing 100084, China; Ministry of Education Key Laboratory of Bioinformatics, Bioinformatics Division at the Beijing National Research Center for Information Science and Technology, Center for Synthetic and Systems Biology, Department of Automation, Tsinghua University, FIT 1-107, Beijing 100084, China; Ministry of Education Key Laboratory of Bioinformatics, Bioinformatics Division at the Beijing National Research Center for Information Science and Technology, Center for Synthetic and Systems Biology, Department of Automation, Tsinghua University, FIT 1-107, Beijing 100084, China; Ministry of Education Key Laboratory of Bioinformatics, Bioinformatics Division at the Beijing National Research Center for Information Science and Technology, Center for Synthetic and Systems Biology, Department of Automation, Tsinghua University, FIT 1-107, Beijing 100084, China

## Abstract

Cell–cell communication (CCC) through ligand–receptor (L–R) pairs forms the cornerstone for complex functionalities in multicellular organisms. Deciphering such intercellular signaling can contribute to unraveling disease mechanisms and enable targeted therapy. Nonetheless, notable biases and inconsistencies are evident among the inferential outcomes generated by current methods for inferring CCC network. To fill this gap, we developed collectNET (http://health.tsinghua.edu.cn/collectnet) as a comprehensive web platform for analyzing CCC network, with efficient calculation, hierarchical browsing, comprehensive statistics, advanced searching, and intuitive visualization. collectNET provides a reliable online inference service with prior knowledge of three public L–R databases and systematic integration of three mainstream inference methods. Additionally, collectNET has assembled a human CCC atlas, including 126 785 significant communication pairs based on 343 023 cells. We anticipate that collectNET will benefit researchers in gaining a more holistic understanding of cell development and differentiation mechanisms.

**Database URL:** http://health.tsinghua.edu.cn/collectnet.

## Introduction

Cell–cell communication (CCC) forms the cornerstone of life activities in multicellular organisms, involving the interaction between ligand–receptor (L–R) pairs [1, 2]. Ligands are proteins that bind to other biomolecules to transmit signals, while receptors are biological molecules that interact with ligands [3]. Binding of ligands to receptors on recipient cell membranes triggers the activation of transcription factors and their target genes within the cell [4, 5]. In this manner, CCC orchestrates processes such as cell proliferation, migration, differentiation, and death, thereby achieving tissue-wide coordination, ultimately resulting in the emergence of complex functionality in cellular organisms. Therefore, deciphering the CCC network plays a pivotal role in advancing the understanding of cell development, tissue homeostasis, immune processes, and further applied to the diagnosis and treatment of major diseases such as cancer [6–8].

Recent advances in single-cell RNA sequencing (scRNA-seq) technology and the accumulation of scRNA-seq data enable the measurement of ligand and receptor expression in single cells across various cell types, thereby assisting in the inference and construction of CCC networks, further enhancing the understanding of human disease mechanisms.

Previous methods employ various approaches to calculate the communication scores between ligand and receptor [9–12]. These methods follow a common computational framework, where feature genes associated with CCC are selected from the matrix. Subsequently, communication scores (i.e. interaction strengths) of L–R pairs are computed. These scores are further utilized to estimate the communication status between cells along each pathway [6, 13]. For example, CellTalker [9] uses ligands and receptors that exhibit non-zero expression in a predefined proportion of cells. CellChat [10] calculates communication scores by multiplying the gene expression of ligands and receptors, which take effects by taking the geometric average of polymer-containing ligands or receptors before the multiplication. CellPhoneDB [11] has proposed enrichment scores, which represent the minimum average gene expression of receptors in specific cell types. Nonetheless, previous benchmarking studies have observed significant differences among the inference results of these methods [14, 15]. For example, LIANA found that regardless of variations in inference methods or reference databases, the overlapped communication pairs remained relatively low within the same dataset [15]. In addition, there remains a deficiency in comprehensive interpretation of the CCC patterns in human organs. While some databases or web servers partially address CCC and provide lists of inferred results [16–20], they often fail to simultaneously support online inference for new data, downstream analysis, and visualization processes while providing a CCC database, which directly leads to limited applications (Supplementary Table S1). Therefore, a comprehensive web platform for inferring, searching, analyzing, and visualizing CCC networks is in pressing need.

**Figure 1.**
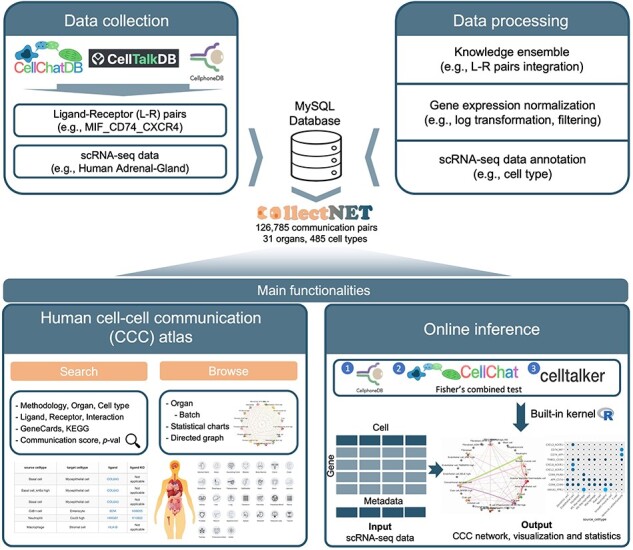
Overview of the collectNET website, an all-in-one web server for integrated inference of CCC network, with efficient calculation, hierarchical browsing, comprehensive statistics, advanced search capabilities, and intuitive visualization.

To circumvent these bottlenecks, we develop collectNET (COmbined ceLL-cEll CommunicaTion NETwork), a comprehensive web-based platform for interpreting CCC network across human organs (Fig. 1). With prior knowledge of 3954 L–R pairs and systematic integration of three mainstream inference methods, collectNET provides an online inference workflow, including data pre-processing, inference, and visualization. Additionally, it provides a CCC atlas spanning human organs, revealing hundreds of thousands of cells and over one hundred thousand significant CCC pairs. The diverse functionalities of collectNET facilitate the identification of topological characteristics and cellular communication pattern features, thereby contributing to further advancements in the investigation of disease etiology and precision medicine research.

## Materials and methods

### Data collection and preprocessing

To fully harness the potential L–R interaction information, we first constructed a comprehensive L–R pair database by integrating three databases, namely CellPhoneDB [11], CellTalkDB [21], and CellChatDB [10]. The integrated L–R database comprises 3954 L–R pairs, including complexes and polymers (Supplementary Table S2). In the construction of the CCC network atlas, we utilized the single-cell gene expression count matrix of 31 human organs from the Human Cell Landscape (HCL) [22]. Then, these datasets underwent preprocessing steps. Firstly, the raw count matrix of gene expression for each dataset is normalized. Here, ${X_{i,j}}$ denotes the expression level of the *i*-th gene in the *j*-th cell from the input raw count matrix, $l{b_j}$ represents the library size of the *j*-th cell, and $s{f_j}$ signifies the size factor for the *j*-th cell. ${X_{norm{\ }i,j}}$ is the normalized expression level of the *i*-th gene in the *j*-th cell.


$$l{b_j} = \mathop \sum \limits_{i = 1}^N {X_{i,j}},{\ }s{f_j} = \frac{{l{b_j}}}{{\overline {lb} }},{\ }{X_{norm{\ }i,j}} = \frac{{{X_{i,j}}}}{{s{f_j}}}$$

Then logarithmic transformation is performed on the normalized gene expression matrix. Here ${X_{log{\ }i,j}}$ is the log-transformed expression level of the *i*-th gene in the *j*-th cell.


$${X_{log{\ }i,j}} = ln\left( {1 + {X_{norm{\ }i,j}}} \right)$$

Subsequently, genes expressed in fewer than three cells and cells expressing fewer than 200 genes were filtered out. It is noteworthy that in the online inference interface, users are afforded the flexibility to customize the parameters according to their preferences.

### Methodology for online inference

We utilized an integration approach to conduct the online inference. Specifically, we employed methodologies for CCC inference from CellPhoneDB [11], CellTalker [21], and CellChat [10], which are widely used and based on differing approaches (Supplementary Text S1). To achieve more precise and reliable construction of the CCC networks, we integrated the results using Fisher’s combined probability [23]. In our integration approach, our goal was to combine both the *P*-value matrix and the communication strength matrix for each single method. The *P*-value matrix indicates the significance of expression for a given L–R pair between two cell types, while the communication strength matrix represents the interaction strength of a specific L–R pair. For the integration of the *P*-value matrix, Fisher’s combined probability test was employed. Specifically, if all the null hypotheses are true and the *P*-value of each single test is independent of each other, the sum of the logarithm of their test statistics follows a chi-square distribution with $2k$ degrees of freedom, where $k$ is the number of tests to be merged.


$$\chi _{2k}^2\sim - 2\mathop \sum \limits_{i = 1}^k ln\left( {{p_i}} \right)$$

In this equation, ${p_i}$ denotes the *P*-value of the *i*-th independent test, with a total number of *k* tests, and *k* is set to 3. Consequently, by integrating three mutually independent hypothesis tests conducted on the interactions between ligands and receptors, we obtained the statistical significance of considering multiple individual experiments. The combined *P*-value for each L–R pair is calculated using the following formula:


$${p_{combined}} = 1 - {F_{\chi _{2k}^2}}\left( { - 2\mathop \sum \limits_{i = 1}^k ln\left( {{p_i}} \right)} \right)$$

Additionally, for the three normalized communication strength matrix, we summed and normalized the corresponding indexed values in the same way to obtain the averaged communication score matrix between cell types.


$${X_{combined{\ }i,j}} = \frac{{\mathop \sum_{k = 1}^{SharedNum}X_{log{\ }i,j}^k}}{{{{\mathop \sum}_{i,j}}\mathop \sum_{k = 1}^{SharedNum}X_{log{\ }i,j}^k}}$$

In this context, the indices *i* and *j* denote the corresponding cell types. The term *SharedNum* denotes the number of times the L–R pair was independently tested in the Fisher’s combined probability test. After normalization, communication scores were obtained for each L–R pair across various cell types. The communication score, a normalized value ranging from 0 to 1, represents the strength of communication between a L–R pair. L–R pairs exhibiting values lower than a specific threshold of 0.05 in the corresponding positions of the *P*-value matrix were filtered out. These L–R pairs are regarded as statistically significant for the respective cell type pairs.

### Web server development

collectNET website is maintained on a Linux-based Apache web server v2.4.58, while optimizing the web interface with the Bootstrap v3.3.7 framework. To implement advanced tables and responsive charts, we utilize a range of jQuery Plugins and JavaScript Libraries. The current version has ensured compatibility with mainstream web browsers such as Google Chrome, Firefox, Opera, Microsoft Edge, Apple Safari, and others.

## Results

### Overview of the functionality of collectNET

collectNET provides a CCC atlas spanning human organs, revealing 126 785 significant communication pairs across 31 organs, 485 cell types, and 343 023 cells. The major functionalities of collectNET encompass online inference, interrogation, visualization, and analysis of CCC network.

#### Online inference and analysis

collectNET allows users to upload their own scRNA-seq data and obtain inferred CCC networks and analyses through its online inference functionality. The results are presented in various formats including tables, circos plots, and bar charts. For online analysis, four widely-used scRNA-seq data formats are accepted: TXT gene expression matrices with CSV annotations, RDS files containing Seurat objects, H5Seurat files, and Anndata-formatted H5AD files. This diverse range of input options ensures compatibility with most single-cell analysis workflows, allowing users to easily upload their data regardless of the computational tools or platforms they employ. Users can also specify the minimum number of genes, minimum number of cells, maximum iterations, number of L–R pairs shown in display, weights for integration, and *P*-value threshold for inference. The corresponding parameter names, descriptions, and default values are provided in Supplementary Table S3. Across different dataset scales, the inference time ranges from several minutes to a dozen minutes (Supplementary Fig. S2). Additionally, collectNET demonstrates reasonable maximum memory usage, enabling concurrent execution of multiple tests and the processing of single-cell datasets comprising up to hundreds of thousands of cells (Supplementary Fig. S3). The inference results include a table containing information on significant L–R pairs, an integrated cell communication circular plot, statistic measures of the cell type information, bar plots of the most frequently occurring ligands and receptors, and heatmaps of L–R pair interactions. In the visualization section, an integrated CCC circular plot is displayed, along with cell type information in the dataset, the top frequently occurring ligands and receptors, and bar plots and heatmaps of L–R pair interactions. We have also meticulously crafted a downloadable tabular format for ease of reference. The rows within this table correspond to the number of significantly expressed L–R pairs between each pair of cell types, as determined by the specified *P*-value threshold. The table is structured with seven columns, which delineate the inference method, task ID, ligand, receptor, receptor–ligand pair, the calculated communication score, and the *P*-value. Notably, both the ligand and receptor entries are hyperlinked to the respective GeneCards entries [24].

#### Advanced searching

collectNET offers advanced search capabilities that facilitate comprehensive exploration of cell–cell interactions. Users have the flexibility to index and query the cell–cell interaction pairs using various parameters, such as methods, organs, cell types, receptors, ligands, or L–R interactions. collectNET supports multi-criteria indexing, allowing for nuanced searches that can be further refined based on the specific research interests of the user. The search results can be sorted according to the *P*-value of the integration method or communication score, providing users with a ranked list of interactions that are statistically significant or biologically relevant. The search results are also downloadable, enabling users to analyze and integrate the data into their own research workflows seamlessly.

#### Intuitively browsing

collectNET provides users with a hierarchical browsing page based on organs and batches, including L–R pairs and rich statistical charts. At the *Home* page, icons representing various organs are accessible, and users can delve into detailed cellular landscapes with a single click. For each organ, collectNET presents a nuanced depiction of cellular composition through pie charts that enumerate the cell type distribution for different organs. Additionally, collectNET provides a detailed account of the most frequently occurring ligands, receptors, and L–R pairs, as identified by our ensemble method, along with their respective frequencies, in a tabular format that underscores the significance of these interactions at the *Browse* page.

Each organ may encompass one or multiple batches, with each batch representing a distinct dataset. collectNET further enriches the exploration of the user by incorporating UMAP plots [25], CellChat [10], CellTalker [9], and CellPhoneDB [11] inference results, as well as the outcomes of our proposed integrated CCC inference method. These are presented alongside a comprehensive table of all significant L–R pairs, offering a multifaceted view of cellular interactions.

The communication networks are visualized through a Circos plot that encapsulates the quantity of cells within each cell type, the number of significant pathways between different cell types, and the communication scores. This visual representation not only enhances the user’s understanding of the cellular ecosystem but also facilitates the identification of key communication hubs and potential targets for therapeutic intervention.

### Extensive applications of collectNET

collectNET has demonstrated a diverse range of application scenarios.

#### collectNET demonstrates a diverse range of capabilities in new data mining

**Figure 2.**
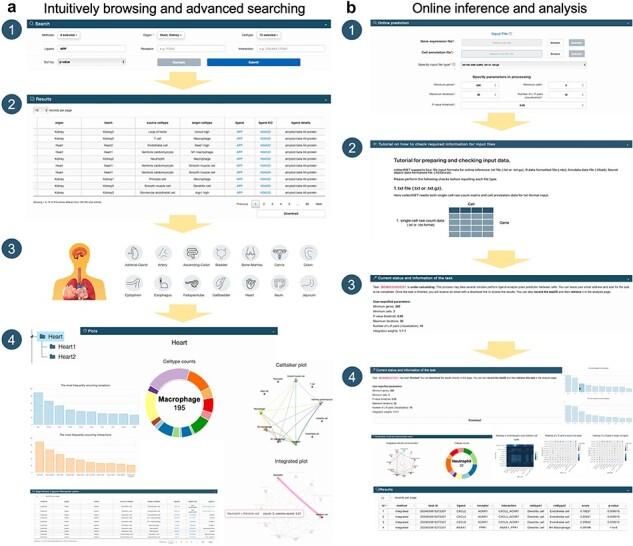
collectNET demonstrates a diverse range of capabilities in new data mining, helping to understand the signaling pathways in which this gene plays a role, the human organs in which it is most likely involved in CCC, and the nature of its function.

If a researcher aims to explore the CCC signaling pathways in which a specific gene is involved, as well as the likelihood of its involvement in CCC across human organs, collectNET can substantially aid in this endeavor (Fig. 2). Specifically, the researcher can first select the name of the ligand or receptor and sort the search results by inference *P*-value or communication score in descending order on the *Search* page. Then, from the search outcomes, the researcher can discern the significant CCCs associated with the gene, occurring in various cell types, ranked by communication strength or confidence level. The researcher can access additional information such as annotations for each L–R pair, along with other pertinent details. For example, if the researcher has a particular interest in the communication status within human heart, the *Browse* page of collectNET articulates a comprehensive array of cell types encapsulated within that organ. Predominantly, CD74 emerges as the most frequently occurring ligand, with an incidence of 142 occurrences, while MIF stands out as the recurrent receptor, noted 89 times. The most prevalent L–R pair is identified as CD74_MIF. Within the cardiac dataset, delineated into two distinct batches, one can procure the inferential outcomes garnered from three disparate methodologies, alongside the visualizations of inferences culled from collectNET, which articulate the count of significant L–R pairs among various cell types in conjunction with the composite communication scores. An explication of the more intricate details of the graph is furnished in the tabular form beneath the visualizations.

#### collectNET reveals the topological characteristics of communication networks

If users aim to explore the internal structure of communication networks, this can be achieved by exploring the topological characteristics of the network by focusing on the macro-level analysis of cell type clustering and specificity. Specifically, we employed graph clustering techniques, where the input for the clustering algorithm was a CCC network matrix computed by collectNET. The PageRank scores for each cell type, treated as nodes, were obtained using the PageRank algorithm (Supplementary Text S2). In the tutorial dataset of 1308 cardiac cells [22] provided in collectNET (Supplementary Fig. S1), the ventricle cardiomyocyte exhibits a PageRank score approaching 0.2, denoting a pronounced importance in the tutorial’s cardiac data. This corresponds to the high attention paid to the ventricle cardiomyocytes as a specialized class of myocytes that are uniquely found within the ventricles of the heart [26]. In contrast, dendritic cells, endothelial cells, and neutrophils all exhibit significantly lower scores, suggesting a more singular signaling pathway presence within these cell types. Also, ventricle cardiomyocytes, macrophages, fibroblasts, smooth muscle cells, M1 macrophages, and M2 macrophages can be identified as the largest clique within the communication network. This observation suggests that these cell types may share a substantial number of common communication patterns, exhibit highly active interactions among each other, and should be comprehensively analyzed within the entire clique for communication studies. Additionally, by performing graph clustering on the communication network given by collectNET, it is evident that, in addition to the aforementioned largest clique, two distinct clusters have emerged: one comprising endothelial cells and neutrophils, and the other dendritic cells. The discovery can be justified since both endothelial cells and neutrophils are associated with the vascular and blood systems, interacting closely in inflammation and immune responses, such as the release of inflammatory mediators and neutrophil migration [27, 28]. This clustering approach provides insights into the functional relationships and potential collaborative roles of these cell types in the context of cardiovascular and immunological processes.

#### collectNET corroborates the efficacy of the inference methodology through statistical methods

**Figure 3.**
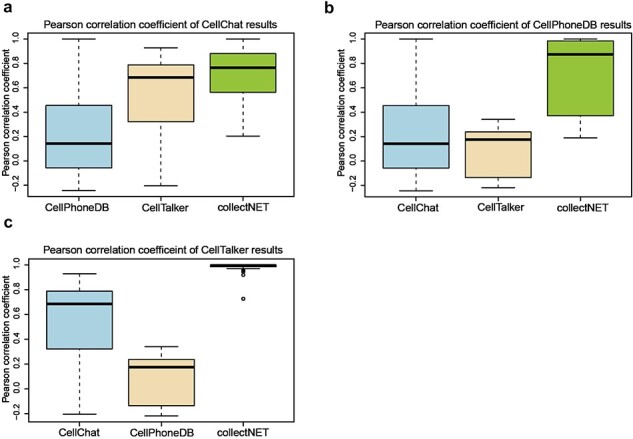
collectNET corroborates the efficacy of the inference methodology through statistical methods, with the *x*-axis representing the three inference methods being compared to the method mentioned in the title and the *y*-axis denoting the PCC, as shown in (a) boxplot of the PCC between the CellChat method and the other methods, (b) boxplot of the PCC between the CellPhoneDB method and the other methods, and (c) boxplot of the PCC between the CellTalker method and the other methods.

Owing to the integration of three distinct inference methods in collectNET, we can empirically assess the significance of this integration through statistical methods. We calculated Pearson correlation coefficients (PCC) on the communication networks inferred by the four methods across 10 independent single-cell datasets from HCL (Fig. 3). Figure 3a presents a boxplot of the PCC between CellChat and the other methods, with the median value of collectNET 0.77 and markedly higher than the other methods. This indicates a strong correlation between the results of collectNET and those of CellChat, surpassing the correlations observed between CellChat and the other two methods. Similarly, Fig. 3b and c demonstrate that the results of collectNET are highly correlated with CellPhoneDB and CellTalker, with the median value of 0.87 and 0.99, respectively. Three one-sided Wilcoxon signed-rank tests were conducted on the PCCs of collectNET and the second-ranked method. *P*-values of the three tests are 0.006, less than 1e-16 and less than 1e-16 respectively, which means that the overall PCC of collectNET is significantly higher than that of each of the second-ranked methods. This underscores that despite the obvious differences between the networks inferred by different methods, collectNET effectively integrates the methods based on different models and databases of L–R pairs, constructing a more accurate communication network by combining diverse biological information.

## Conclusion

CCC is accomplished through L–R pairs based intercellular signaling. The study of CCC is of great significance as it allows for the investigation of fundamental biological questions, such as development and differentiation, the understanding of how overall tissue coordination and functionality are achieved, as well as the analysis of the coordinated actions of multiple cells in disease onset. Currently, various methods have been developed to infer CCC from single-cell transcriptomic sequencing data, but significant biases and inconsistencies in the inference results of these methods remain to be shown. In addition, existing databases or web servers often fail to simultaneously support online inference for new data and related analyses. collectNET highlights multiple notable features. collectNET integrates CellChat [10], CellPhoneDB [11], and CellTalker [9] based on Fisher’s combined probability test, to enhance the reliability of intercellular communication inference, while incorporating three widely-used databases of ligand–receptor pairs as prior knowledge to extract maximum information from the input data. The platform enables users lacking coding expertise or computational resources to perform efficient inference and visualization through intuitive tutorial-style interfaces. collectNET provides downloadable tables for diverse methods, organs, and user-submitted tasks, supporting versatile applications like drug target prediction and immunotherapy strategies. It furnishes extensive CCC resources, encompassing 126 785 significant communication pairs on 31 organs and 485 cell types based on the scRNA-seq datasets of 343 023 single cells.

## Discussion

In future version, collectNET will undergo substantial refinements across two primary dimensions. Firstly, we will maintain synchronicity with the latest advancements in CCC inference methodologies to ensure the comprehensiveness and precision of the inference outcomes. Secondly, the incorporation of a diverse spectrum of omics data, encompassing single-cell spatial omics data, will be meticulously contemplated. This multifaceted approach will seamlessly introduce coordinate information into the inference of CCCs, thereby elevating the accuracy and robustness of the derived interactions. Such an expansion of data sources is poised to yield a more nuanced and contextually grounded portrayal of the intricate cellular dialogues within the biological system. We anticipate that collectNET will bring substantial benefits to both biologists and algorithm researchers, enabling them to illuminate cellular differentiation, unravel developmental mechanisms, and pioneer innovative strategies for disease treatment with unprecedented clarity and insight.

## Supplementary Material

baae098_Supp

## Data Availability

The source code for online analysis, associated applications, and the corresponding Docker file are available at https://github.com/wcanisay-y/collectNET.
