# Supplementary material for: collectNET: a web server for integrated inference of cell–cell communication network
Source: Database (Oxford). 2024 Sep 16;2024:baae098. doi: 10.1093/database/baae098 (PMC11403813; doi:10.1093/database/baae098)
Supplement: baae098_Supp [file baae098_supp.zip › suppl_data/Supplementary_info_clean_submit.docx]

**Supplementary Information**

**collectNET: a web server for integrated inference of cell-cell communication network**

Yan Pan1,#, Zijing Gao1,#, Xuejian Cui1, Zhen Li1 and Rui Jiang^1,*^

^1^ Ministry of Education Key Laboratory of Bioinformatics, Bioinformatics Division at the Beijing National Research Center for Information Science and Technology, Center for Synthetic and Systems Biology, Department of Automation, Tsinghua University, Beijing 100084, China

# These authors are equal contributors.

* To whom correspondence should be addressed.

E-mail: ruijiang@tsinghua.edu.cn

**Contents**

[Supplementary Texts 3](#_Toc173317319)

[Text S1. Methodology of collectNET online inference 3](#_Toc173317320)

[Text S2. Calculation of PageRank score 5](#_Toc173317321)

[Supplementary Figures 6](#_Toc173317322)

[Figure S1. collectNET reveals the topological characteristics of communication networks. 6](#_Toc173317323)

[Figure S2. Computational efficiency of collectNET. 7](#_Toc173317324)

[Figure S3. Memory usage of collectNET. 8](#_Toc173317325)

[Supplementary Tables 9](#_Toc173317326)

[Table S1. Comparison of collectNET with other published cell-cell communication databases or websites 9](#_Toc173317327)

[Table S2. Comparison of collectNET and other reference ligand-receptor pair databases 10](#_Toc173317328)

[Table S3. The user-defined parameters for collectNET. 11](#_Toc173317329)

[References 12](#_Toc173317330)

**Supplementary Texts**

**Text S1. Methodology of collectNET online inference**

In collectNET, we selected three of the most influential, widely-used, and highly-cited cell-cell communication inference methods for integration, namely CellChat [1], CellPhoneDB [2], and CellTalker [3]. The rationale for employing multiple methods lies in their differing inferential strategies and methodologies, despite all leveraging known ligand-receptor pair information to analyze intercellular interactions based on single-cell RNA sequencing data. Specifically, CellChat employs a probabilistic model to infer cell-cell communication, considering not only ligand-receptor pairs but also multimeric complexes and co-factors, thereby enabling the simulation of more complex cellular interactions at the pathway level. CellPhoneDB utilizes permutation tests to assess interaction significance, focusing primarily on direct ligand-receptor interactions while accounting for protein complex structures. Conversely, CellTalker focuses on the expression patterns of known ligand-receptor pairs within and between cell populations. Each method thus offers distinct advantages: CellChat considers the role of additional biomolecules in cell-cell communication, CellPhoneDB emphasizes statistical rigor and robustness, and CellTalker provides a straightforward approach for evaluating intercellular communication. Therefore, we aim to integrate the results inferred by these methods and deploy them on the website to provide online inference services, enabling users to obtain more comprehensive and reliable cell-cell communication networks.

To integrate these tools, we employed a statistical approach known as Fisher's combined probability test. In this test, if all the null hypotheses are true and the *p*-values of each single test are independent, the sum of the logarithms of their test statistics follows a chi-square distribution with 2𝑘 degrees of freedom, where 𝑘 is the number of tests to be merged. In the implementation of collectNET, by integrating three mutually independent hypothesis tests conducted on the interactions between ligands and receptors, we obtained the statistical significance of considering multiple individual experiments. This approach offers several significant, including enhanced robustness by combining *p*-values from multiple methods, which mitigates biases or limitations inherent to any single approach. It also increases sensitivity, enabling the detection of significant interactions that might be overlooked by individual methods, and reduces false positives, as interactions consistently identified across methods are more likely to represent genuine biological phenomena. Additionally, this integrative approach leverages the unique strengths of each method, potentially yielding a more comprehensive and nuanced picture of cell-cell communication.

In conclusion, by harnessing the complementary strengths of CellChat, CellPhoneDB, and CellTalker through Fisher's combined probability test, our website can provide assurance for comprehensive, accurate, and robust inference of cell-cell communication.

**Text S2. Calculation of PageRank score**

As an algorithmic measure of the relative importance of a node within a network, the PageRank score is used to explore the role assumed by each node [4]. The PageRank algorithm can be expressed by the following formula for iterative computation.

$$P_{i}^{\left( t+1 \right)}=\alpha\sum_{j\in N\left( i \right)} \frac{P_{j}^{\left( t \right)}/L_{j}}{N_{j}}+\left( 1-\alpha\right)\frac{1}{N}$$

where $P_{i}^{\left( t \right)}$​ is the PageRank value of cell type *i* at iteration *t*, $\alpha$ is the damping factor set to 0.85, $L_{j}$​ is the total number of outgoing links from cell type *j*, and $N$ is the total number of cell types in the network. At the start of the iterative algorithm, the PageRank value of each node is set to $1/N$. The iteration stops after multiple iterations when the change in PageRank values between two consecutive iterations is less than a certain threshold, which is set at 1% of the initial value. Within communication networks provied by collectNET in the tutorial data, the PageRank score quantifies the significance of interactions within cell type pairs throughout the biological process.

**Supplementary Figures**

**Figure S1.** **collectNET reveals the topological characteristics of communication networks.**

**
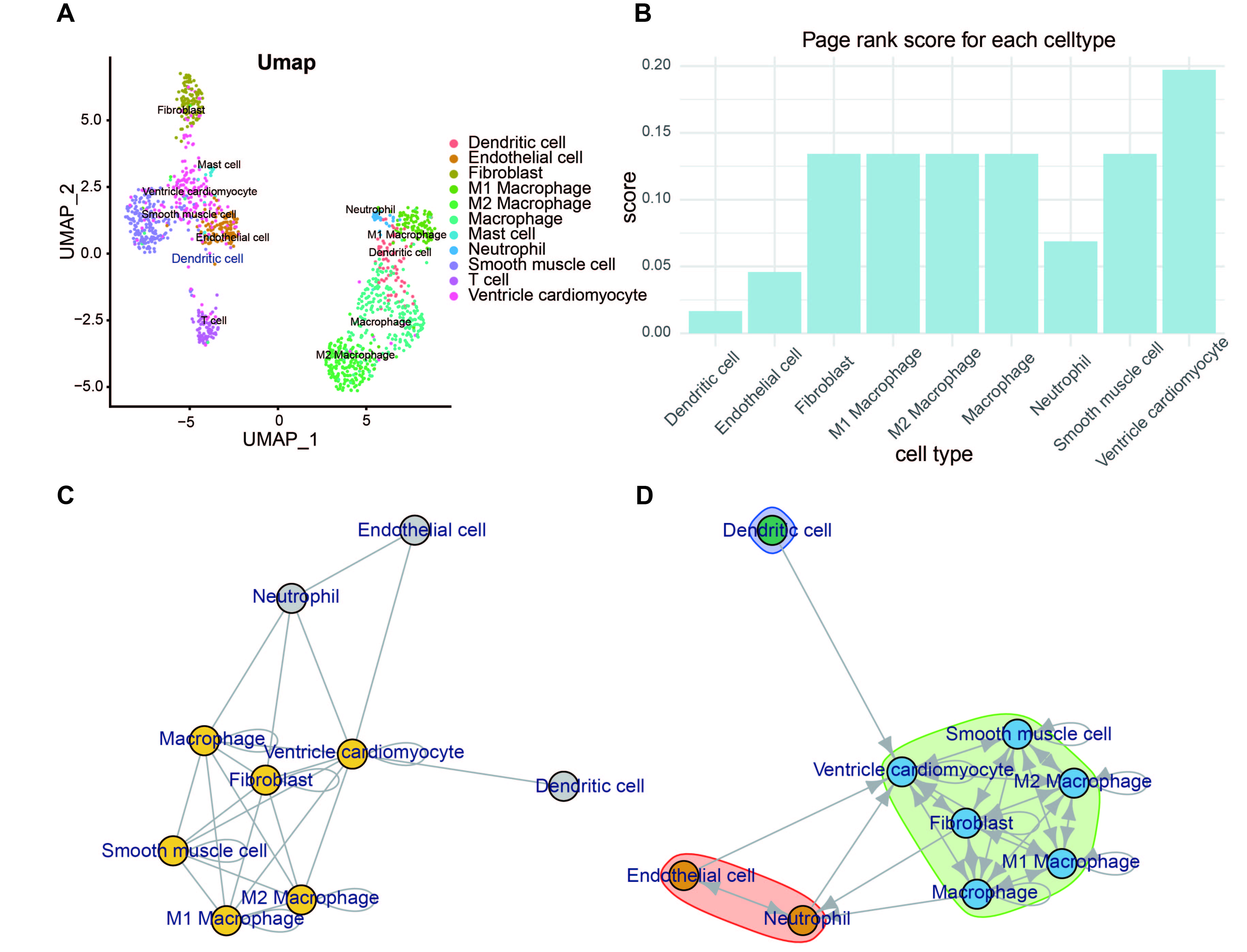
**

**Figure S1. collectNET reveals the topological characteristics of communication networks. (**A) Umap plot on tutorial data. (B) PageRank score quantifies the significance of each cell type's interactions with others throughout the biological process. (C) The largest clique found by using graph search algorithms. (D) The graph clustering based on strong connectivity. Both (C) and (D) provide insights into the functional relationships and potential collaborative roles of these cell types.

**Figure S2. Computational efficiency of collectNET.**

**
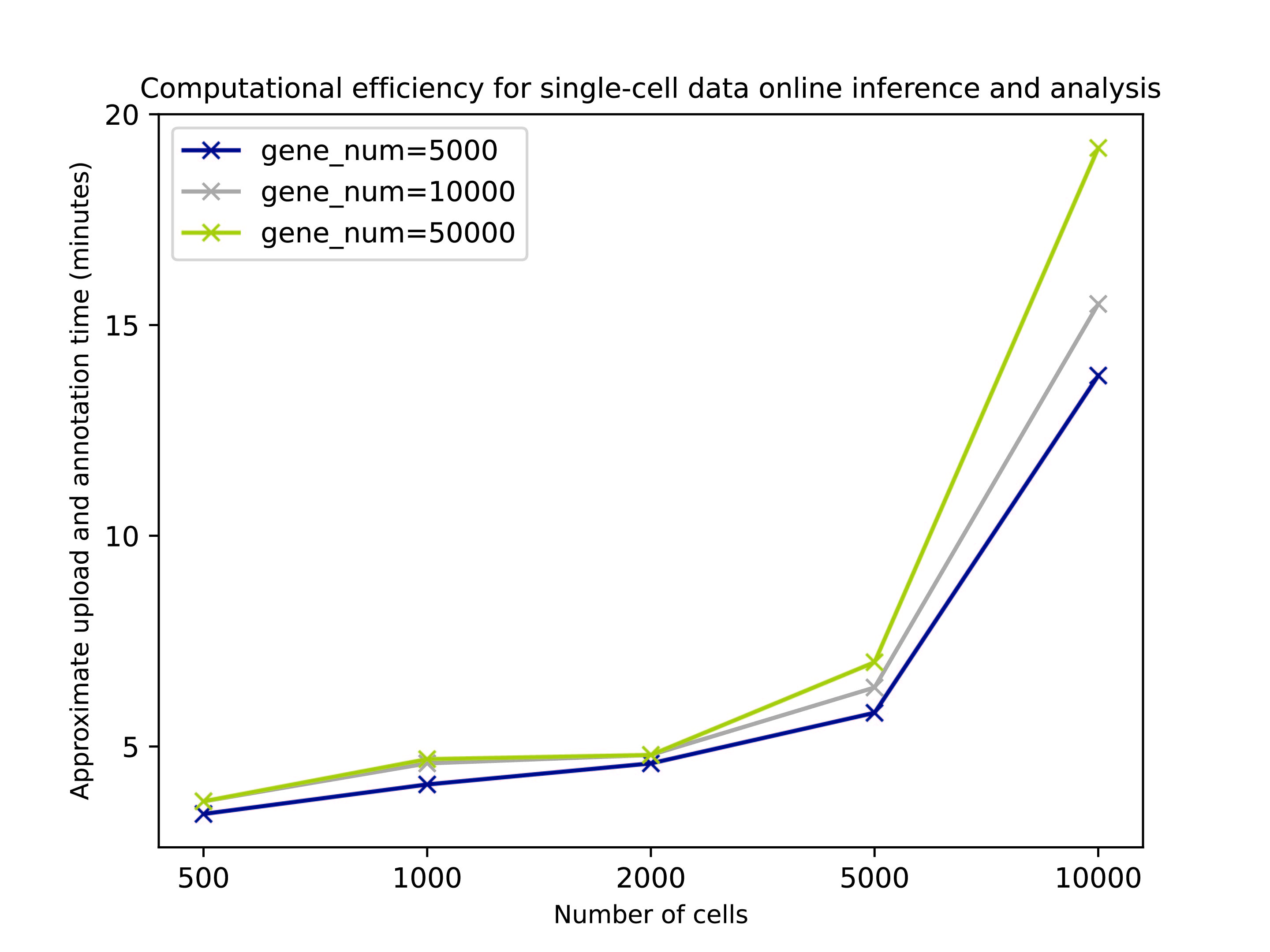
**

**Figure S2. Computational efficiency of collectNET.** Computational efficiency for single-cell data online inference and analysis, where the x-axis denotes the number of cells ranging from 500 to 10,000, and the y-axis denotes approximate upload and annotation time (minutes).

**Figure S3. Memory usage of collectNET.**

**
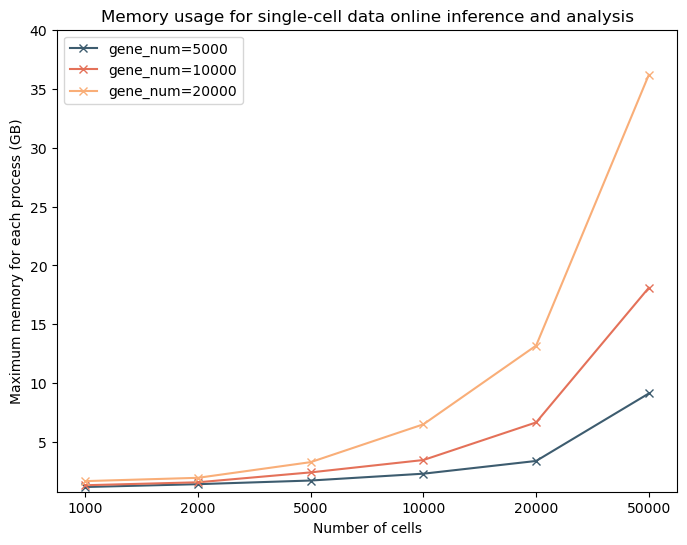
**

**Figure S3. Memory usage of collectNET.** Maximum memory usage for single-cell RNA-seq data online inference and analysis, where the x-axis denotes the number of cells ranging from 1,000 to 50,000, and the y-axis denotes the peak memory usage for each task.

# **Supplementary Tables**

**Table S1.** Comparison of collectNET with other published cell-cell communication databases or websites

| **Categories** | **Functionalities and applications** | **collectNET** | **CellCommuNet [5]** | **CITEdb [6]** | | **TALKIEN [7]** |
| --- | --- | --- | --- | --- | --- | --- |
| Prior data | Integration of various L-R pair databases | ✓ | ✓ |  | ✓ | |
| Methods for inference | CellChat | ✓ | ✓ |  |  | |
|  | CellPhoneDB | ✓ |  |  |  | |
|  | CellTalker | ✓ |  |  |  | |
| Database | Diverse forms of statistical graphs | ✓ | ✓ | ✓ | ✓ | |
|  | Multiple externel hyperlinks | ✓ | ✓ | ✓ | ✓ | |
|  | Multi-tiered Search and Download funtionality | ✓ | ✓ | ✓ |  | |
| Web service | Support for user-provided single-cell data | ✓ | ✓ |  |  | |
|  | Innovative inference methology | ✓ |  |  |  | |
|  | Customizable parameters for network construction | ✓ |  |  |  | |
|  | Downstream analysis of the network | ✓ |  |  | ✓ | |

**Table S2.** Comparison of collectNET and other reference ligand-receptor pair databases

| **L-R Databases** | **L-R pairs** | **Contain Copolymers** |
| --- | --- | --- |
| collectNET | 3954 | Yes |
| CellChatDB | 1939 | Yes |
| CellPhoneDB | 1396 | Yes |
| CellTalkDB | 2557 | No |

**Table S3.** The user-defined parameters for collectNET.

| **Parameters** | | **Description** | |
| --- | --- | --- | --- |
| Gene expression file | | The input single-cell RNA sequencing matrix file, both txt and rds formats supported. | |
| Cell annotation file | | The input single-cell data cell labels, csv format supported. | |
| Specify input file type | | The format of the input file. | |
| Minimum genes | | The minimum number of genes required per cell (for cell filtering). | |
| Minimum cells | | The minimum number of cells in which a gene must be expressed (for gene filtering). | |
| Maximum iterations | | The number of iterations for inference in CellPhoneDB and CellTalker methods. | |
| Number of L-R pairs (visualization) | | The most frequently occurring ligand-receptor pairs and their quantities displayed in the result visualization. | |
| *p*-value threshold | | The threshold for L-R pairs considered significant in the inference results. | |
| Weight for integration | CellChat | | The weight of communication values in the CellChat method within the integrated approach. |
|  | CellPhoneDB | | The weight of communication values in the CellPhoneDB method within the integrated approach. |
|  | CellTalker | | The weight of communication values in the CellTalker method within the integrated approach. |

# **References**

1. Jin, S., et al., *Inference and analysis of cell-cell communication using CellChat.* Nature communications, 2021. **12**(1): p. 1088.

2. Efremova, M., M. Vento-Tormo, S.A. Teichmann, and R. Vento-Tormo, *CellPhoneDB: inferring cell–cell communication from combined expression of multi-subunit ligand–receptor complexes.* Nature protocols, 2020. **15**(4): p. 1484-1506.

3. Cillo, A.R., et al., *Immune landscape of viral-and carcinogen-driven head and neck cancer.* Immunity, 2020. **52**(1): p. 183-199. e9.

4. Page, L., S. Brin, R. Motwani, and T. Winograd, *The PageRank citation ranking: Bringing order to the web*. 1999, Stanford infolab.

5. Ma, Q., Q. Li, X. Zheng, and J. Pan, *CellCommuNet: an atlas of cell–cell communication networks from single-cell RNA sequencing of human and mouse tissues in normal and disease states.* Nucleic Acids Research, 2024. **52**(D1): p. D597-D606.

6. Shan, N., et al., *CITEdb: a manually curated database of cell–cell interactions in human.* Bioinformatics, 2022. **38**(22): p. 5144-5148.

7. Moratalla-Navarro, F., V. Moreno, and R. Sanz-Pamplona, *TALKIEN: crossTALK IntEraction Network. A web-based tool for deciphering molecular communication through ligand–receptor interactions.* Molecular Omics, 2023. **19**(9): p. 688-696.
